# A rare acantholytic variant of squamous cell carcinoma of the maxilla: A case report and literature review

**DOI:** 10.1097/MD.0000000000021631

**Published:** 2020-08-07

**Authors:** Jo-Eun Kim, Chena Lee, Kyu-Young Oh, Kyung-Hoe Huh

**Affiliations:** a Department of Oral and Maxillofacial Radiology, Seoul National University Dental Hospital; b Department of Oral and Maxillofacial Radiology, Yonsei University College of Dentistry; c Department of Oral Pathology, School of Dentistry, Seoul National University; d Department of Oral and Maxillofacial Radiology and Dental Research Institute, School of Dentistry, Seoul National University, Seoul, Republic of Korea.

**Keywords:** magnetic resonance imaging, prognosis, squamous cell carcinoma of head and neck, tomography, x-ray computed

## Abstract

**Rationale:**

Acantholytic squamous cell carcinoma (ASCC) is an uncommon histopathologic variant of squamous cell carcinoma (SCC), which is the most common malignancy of the oral cavity. Though ASCC showed poor prognosis, the exact diagnosis is challenging.

**Patients concerns:**

A 59-year-old female patient with 1-month long symptoms of pain and burning sensation in the right maxilla.

**Diagnoses:**

Incisional biopsy in the maxilla established the pathologic diagnosis of SCC.

**Intervention:**

The patient underwent mass resection with near total maxillectomy.

**Outcomes:**

The final diagnosis through the microscopic examination was ASCC. Palliative chemotherapy was done to relive the symptoms after the recurrence, however, the patient died of the disease at 8 months after her initial presentation.

**Lessons:**

Special attention should be paid to this variant of SCC because most patients with ASCC have a very poor prognosis.

## 1 Introduction

Squamous cell carcinoma (SCC) is the most common malignancy encountered in the oral cavity and has several histopathological variants. Acantholytic SCC (ASCC) is an uncommon histopathologic variant of SCC, which shows extensive acantholysis of malignant epithelial cells, resulting in pseudovascular or pseudoglandular appearance. Thus, it is also known as adenoid SCC, pseudoglandular SCC, SCC with gland-like features, angiosarcoma-like SCC, and pseudovascular adenoid SCC.^[1]^ It was first described in 1947, as a cutaneous adenoacanthoma that originates from sweat glands;^[2]^ however, subsequent studies revealed that the lesion had a non-eccrine origin. Most of reported ASCC cases were arising from the skin, especially in the sun-exposed region.^[3]^ Occurrences in upper aerodigestive mucosa including lip, oral cavity, nasopharynx, and larynx has been reported, and the first reported case in the oral cavity mucosa was in 1977, by Goldman et al.^[4]^ However, ASCC of oral cavity were extremely rare with only 18 reported cases. The differential diagnosis for intraoral ASCC includes adenosquamous, adenoid cystic, and mucoepidermoid carcinomas of minor salivary gland origin, as well as metastatic adenocarcinoma and angiosarcoma.^[5]^

Here we report the clinicoradiological features of a case with ASCC in the maxilla, and review the available literature to reveal the prognosis of this cancer variant.

## 2 Case presentation

Ethical approval was waived by the institutional review board of our hospital (ERI19042), because this study was a retrospective case report and the patient was passed away. The husband of the patient, her legal representative, provided the informed consent to report the case.

**Figure 1:**
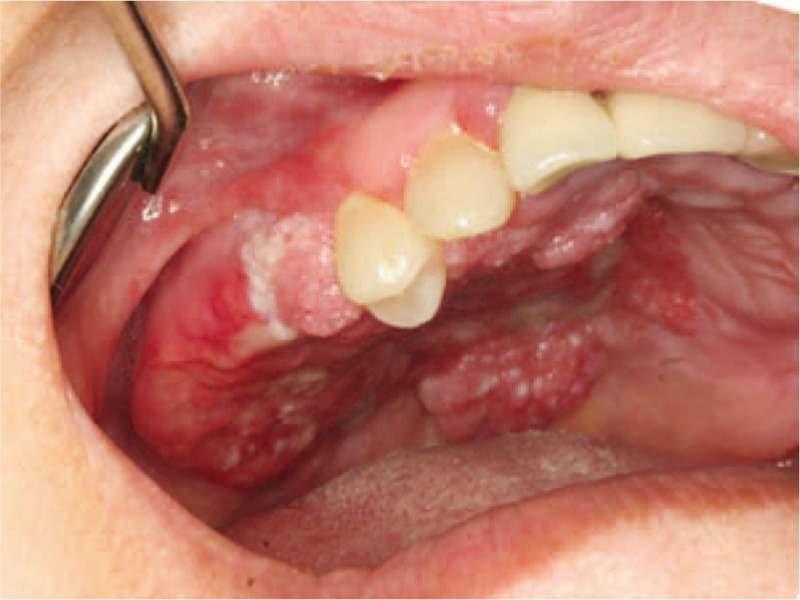
Photographic image of the patient with a large mass having a verrucous surface and ulceration on the right maxillary gingiva and palate.

A 59-year-old female patient with 1-month long symptoms of pain and burning sensation in the right maxilla visited the department of oral medicine at our hospital. Clinical examination revealed an irregular hypertrophic lesion with a verrucous erythematous surface and ulceration on the right palate and edentulous gingiva of the right maxilla (Fig. 1). The pathology result of an incisional biopsy sample reported SCC and recommended re-sampling from a deeper portion of the lesion. The patient was referred to the department of oral and maxillofacial surgery where cancer work-up was performed for surgery.

**Figure 2:**
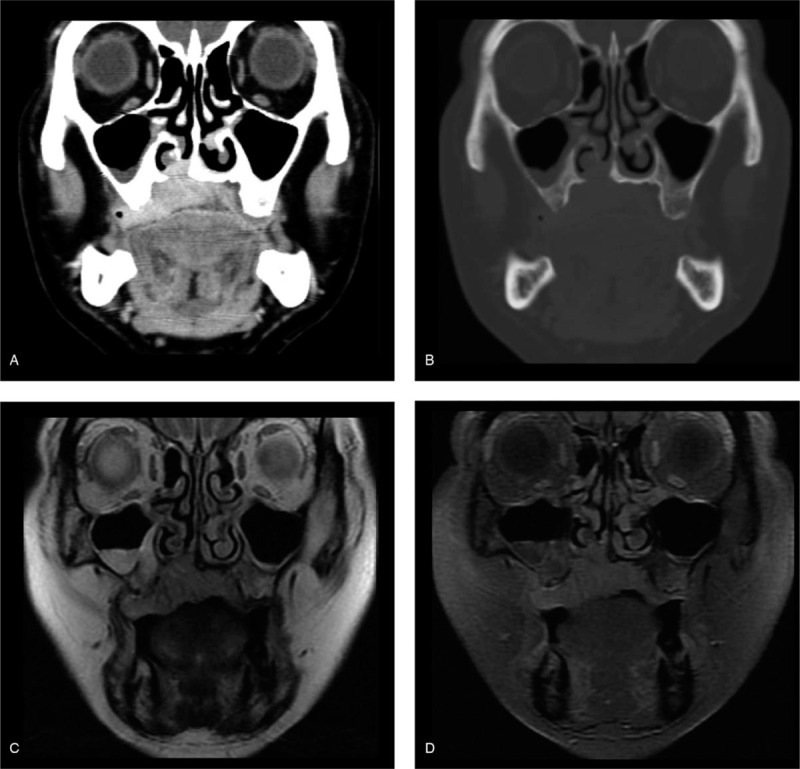
Radiologic images showing an enhancing mass on the palate, destructing the palatine process of the maxilla and infiltrating into the nasal cavity. Coronal images of contrast-enhanced CT with soft tissue window (a), with bone window (b), T2-weighted MR (c), and postcontrast MR (d).

Computed tomography (CT) and magnetic resonance (MR) images revealed a well enhancing mass of approximately 38 × 34 × 18 mm in the right palate (Fig. 2). While destructing the inferior aspect of the nasal cavity, the enhancing mass extended across the midline along the palatal mucosa. The greater palatine foramen was occupied by the mass, but evidence of perineural spread into the pterygopalatine fossa was not found on CT and MRI. No remarkable cervical lymph nodes were observed.

**Figure 3:**
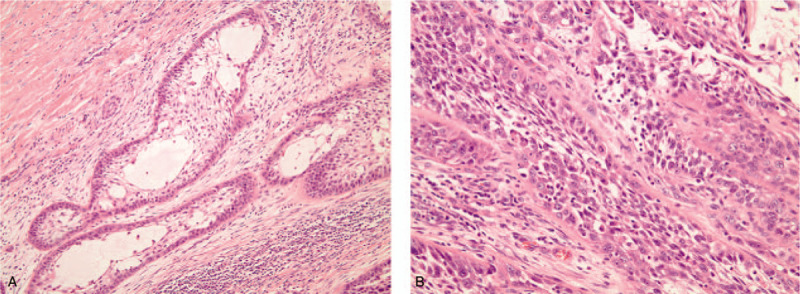
Microscopic images showing acantholysis of malignant squamous cells forming pseudoluminar or pseudoglandular structures [hematoxylin and eosin (a) original magnification × 100, (b) original magnification × 200].

**Figure 4:**
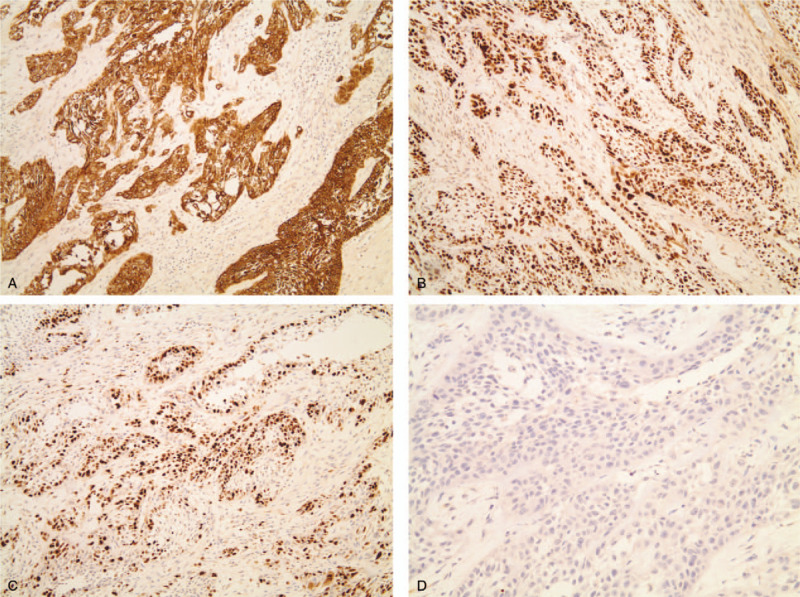
Microscopic images showing immunohistochemistry result of acantholytic squamous cell carcinoma. Tumor cells are immunoreactive for (a) pancytokeratin and (b) p53 (original magnification × 100). (c) A high Ki-67 proliferative index is observed (original magnification × 100). (d) Tumor cells showing pseudoglandular appearance are negative for cytokeratin 7 (original magnification × 200).

The patient underwent mass resection with near total maxillectomy, and the maxilla was reconstructed using a radial forearm free flap. The total specimen was sent to the department of oral pathology. Histopathologic examination revealed clear margins of tumor cells. Nests of malignant squamous cells were identified and showed marked acantholysis giving rise to anastomosing spaces with pseudoglandular appearance (Fig. 3). Immunohistochemical examinations were performed to precisely diagnose the lesion (Fig. 4). Most neoplastic epithelial cells were marked with pancytokeratin and p53, and the strong staining for Ki-67 represented the high proliferative activity of the tumor cells. Although pseudoluminar spaces mimicking adenoid features of a salivary gland tumor made the differential diagnosis difficult, the neoplastic cells did not react with cytokeratin (CK) 7. Based on all the above findings, a diagnosis of ASCC was made.

**Figure 5:**
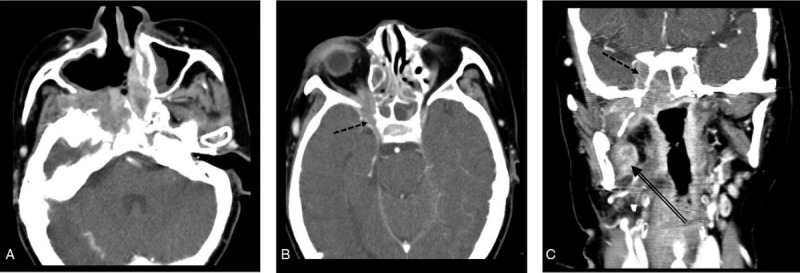
CT images of recurrent lesion. (a) A recurrent lesion destructing the cranial base adjacent to the upper resection margin of the previous surgery. Note the intracranial invasion into the right cavernous sinus through perineural spread (b and c, dotted arrow). Another recurrent lesion in the medial pterygoid muscle (c, double arrow).

Six weeks after the surgery, the patient underwent postoperative radiotherapy (63 Gy, 28 cycles) for a month. Three weeks after the radiotherapy, she visited our hospital with complaints of ocular pain and swelling on the right side. Physiologic examination revealed limitation of ocular movements and ptosis of the right upper eyelid. Subsequent CT revealed extensive recurrent masses in the right pterygoid muscle and pterygopalatine fossa, destruction of the adjacent cranial base with extension into the cavernous sinus, and superior orbital fissure (Fig. 5). Palliative chemotherapy was initiated to relive the symptoms; however, the patient died of the disease at 8 months after her initial presentation.

## 3 Discussion

As a variant of SCC, ASCCs of the oral cavity are extremely rare and need to be differentiated from adenosquamous carcinoma, adenoid cystic carcinoma, mucoepidermoid carcinoma of minor salivary gland origin, and metastatic adenocarcinoma and angiosarcoma.^[5]^ Microscopical features of ASCC include a nonsolid component that contains single or grouped acantholytic and dyskeratotic epithelial cells or cellular debris under the conventional squamous cells, and this nonsolid component forms pseudoglandular or pseudovascular structures. Markers useful for tumor differentiation include the CK7+/CK20− for salivary duct cells, which are negative in SCC cells,^[6]^ and vascular markers, which help distinguish angiosarcomas, although they can appear histologically similar.

Our case exhibited a large recurrent tumor in just 2 months after surgical treatment, with intracranial invasion through perineural spread. The initial diagnostic images and pathological examination from preoperative biopsy presented no remarkable features to help us differentiate the lesion from conventional SCC, and the resection margins around the tumor specimen after the surgical procedure were clear of tumor cells. However, local recurrence occurred very rapidly and widely despite postoperative radiotherapy, and the patient died of the disease at 8 months after her initial presentation. Our case with ASCC represented a very grave prognosis.

ASCC has a poorer prognosis than conventional SCC, with a more aggressive behavior and a greater risk for local recurrence and metastasis.^[2]^ However, because most reports regarding ASCC have been tumors of sun-exposed skin, little is known about the behavior of this variant of oral ASCC, and its prognosis in mucosal sites is controversial. We reviewed English-language literature on PubMed regarding ASCC in the oral cavity. Lesions on the lip vermilion, which can be detected and treated earlier, were excluded from our review. Nineteen ASCC cases in the oral cavity, including the present case, have been reported (Table 1).^[4–8,10–18]^ The average age of the patients was 58 years (range, 38 to 86 years). Of 19 patients, 8 were women and 11 were men. Ten cases had lesions located in the gingival mucosa, 5 in the tongue, 3 in the mouth floor, and 1 in the buccal mucosa. Except in 5 cases for which clinical features were not described, the most frequent clinical characteristic was an ulcerative mass. We found follow-up records of 11 reports, of which 8 had local recurrences developed at 1 to 26 months (mean, 8.4 months) after treatment and died of disease at 3 to 46 months (mean, 12.3 months) after the initial diagnosis (73%; 8 of 11 cases). One of the 8 recurrent cases showed metastasis into the lymph nodes at 1 month after surgery. Although the prognosis for patients with mucosal ASCC remains controversial, our review of cases suggests that this variant of cancer is more aggressive and has a poorer prognosis than conventional SCC.^[7]^ Alterations in the expression of molecules such as E-cadherin and B-carotene, which mediate cell–cell and cell–extracellular matrix adhesions, have been associated with acantholysis and the aggressive behavior of ASCCs.^[8–10]^

**Table 1:**
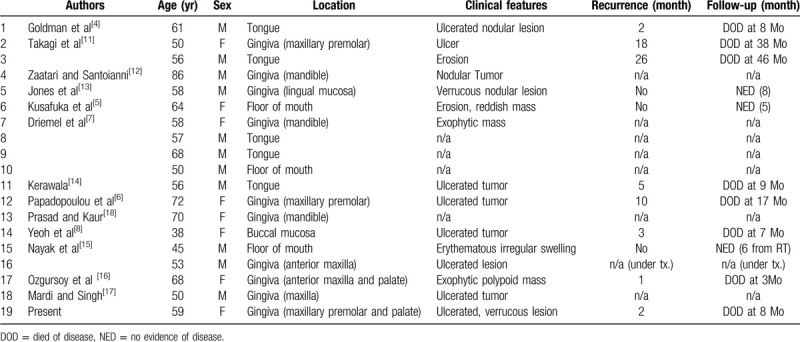
Summary of reported cases of acantholytic squamous cell carcinoma in the oral mucosa.

Diagnosing ASCC before performing a thorough pathological examination is difficult because no specific macroscopic features have been identified, and no pathognomonic imaging features are observed. Our literature review and our experience with the present case revealed a more aggressive behavior and poorer prognosis for oral cavity ASCCs. With a diagnosis of ASCC, the clinicians should consider an aggressive, multidisciplinary treatment and a close follow-up with multimodality images to improve patient outcomes.

## Author contributions

**Conceptualization:** Jo-Eun Kim, Kyung-Hoe Huh.

**Data curation:** Jo-Eun Kim, Kyu-Young Oh.

**Investigation:** Chena Lee, Kyung-Hoe Huh.

**Methodology:** Jo-Eun Kim, Chena Lee, Kyung-Hoe Huh.

**Resources:** Jo-Eun Kim.

**Supervision:** Kyung-Hoe Huh.

**Validation:** Jo-Eun Kim, Kyu-Yong Oh.

**Writing – original draft:** Jo-Eun Kim.

**Writing – review & editing:** Jo-Eun Kim, Chena Lee, Kyung-Hoe Huh.
